# EEG-based functional connectivity patterns during boredom in an educational context

**DOI:** 10.1038/s41598-025-19245-7

**Published:** 2025-10-10

**Authors:** Rajamanickam Yuvaraj, N. P. Guhan Seshadri, Sampathraman Samyuktha, Jack S. Fogarty, Jun Song Huang, Samuel Tan, Teck Kiong Wong

**Affiliations:** 1 Science of Learning in Education Centre (SoLEC), National Institute of Education, Nanyang Technological University, 1 Nanyang Walk, Singapore, 637616 Singapore; 2 Neurological Institute, Cleveland Clinic, Cleveland, OH 44195 USA; 3 Education Technology Division (ETD), Ministry of Education (MOE), Singapore, 138675 Singapore

**Keywords:** Boredom, Education, EEG, Brain connectivity, Learning, Emotion, Coherence, Emotion, Biomedical engineering

## Abstract

Boredom is a common yet understudied emotional state that can adversely impact cognitive performance, motivation, and mental well-being. Gaining insight into its neural basis is crucial for developing strategies to manage or reduce its impact across various settings, including education contexts. The present study investigated brain functional connectivity during boredom in an educational context using electroencephalography (EEG). It was hypothesized that the brain exhibits distinct connections during the experience of boredom. Eighty-four healthy adults (mean age = 26.90 ± 5.29 years) were asked to watch two educational videos designed to induce boredom or neutral states while their EEG signals were captured. Functional connectivity matrices were constructed using coherence in traditional EEG frequency bands. Clustering coefficient $\:\left({C}_{eff}\right)$, characteristic path length $\:\left({C}_{pl}\right)$, global efficiency $\:\left({E}_{glo}\right)$, local efficiency $\:\left({E}_{loc};\right)$, and node strength $\:\left({N}_{str}\right)$ were calculated to compare scalp-level network characteristics between boredom and neutral states. The results showed significant differences in functional connectivity in the alpha, beta, and gamma bands. Boredom was characterized as having significantly higher $\:{E}_{glo}$ and lower $\:{C}_{pl}\:\:$in the alpha, beta, and gamma bands, relative to the neutral condition. Gamma band $\:{C}_{eff}\:$and $\:{E}_{loc}$ were also found to be higher during boredom. The study findings suggest that boredom is associated with distinct patterns of brain functional connectivity, potentially reflecting increased internal processing. The study findings contribute to the broader field of affective neuroscience by highlighting how boredom can influence functional brain network activity in educational contexts.

**Supplementary Information:**

The online version contains supplementary material available at 10.1038/s41598-025-19245-7.

## Introduction

Boredom is a complex and multifaceted emotional state marked by diminished engagement, interest, or motivation in a given activity or task. In the field of psychology, boredom is recognized as a negative affective or emotional state, primarily due to its association with subjective feelings of dissatisfaction, lack of motivation, and disengagement^1,2^. It is also considered to involve both cognitive and emotional components. Cognitively, boredom involves difficulty maintaining attention and a perceived lack of meaningful engagement; emotionally, it reflects an aversive inner experience that signals a need for change or stimulation^3^. Boredom is most likely to occur when individuals are not able to focus their attention on information in a meaningful way, or on a challenge in their environment, leading to feelings of weariness and dissatisfaction^1^. In educational settings, or the workplace^4^, boredom can be detrimental, as it poses challenges for individuals striving to maintain goal-directed behavior and performance^5^. The causes of boredom are both contextual and individual. Contextual factors include monotonous or repetitive tasks, low cognitive challenge, or unengaging instructional environments^6^. At the same time, on the individual level, personal traits such as low self-regulation, high boredom proneness, and attentional difficulties can increase an individual’s susceptibility to boredom^7^. Despite its prevalence and relevance, boredom has received little attention in scientific research.

In education, boredom has been shown to impact learning negatively. For instance, Westgate and Wilson explored the relationship between boredom, cognitive engagement, and academic performance, finding that boredom was linked to decreased cognitive engagement and lower academic outcomes^8^. Eastwood et al. examined the impact of boredom on memory retention, showing that bored students have difficulty encoding and recalling information, significantly impeding the learning process^1^. Pekrun et al. investigated the long-term effects of boredom on academic performance, revealing that a high susceptibility to boredom correlates with lower grades and academic achievement^9^. In addition, Mann and Robinson studied classroom behaviors associated with boredom, noting an increase in disruptive behaviors among bored students, illustrating the importance of engaging teaching methods to mitigate boredom and promote positive classroom behavior^10^. Meta-analyses also show that boredom is negatively related to student engagement and motivation^11^, with previous research indicating boredom significantly decreases engagement and active participation in learning^12^. All these previous studies of boredom have demonstrated a significant impact on students’ learning outcomes, engagement, motivation, and overall academic performance. To counteract boredom, teachers could create learning environments that use moments of boredom to help students focus better. Giving students chances to explore, solve problems, or think creatively when not engaged could help them find ways to stay interested. Additionally, helping students manage their boredom could help them become more effective learners. For instance, exercises that help students focus or shift their attention could encourage them to view boredom as an opportunity for improving concentration and learning, instead of just a sign of disinterest.

Electroencephalography (EEG) has shown strong potential in studying cognitive functions, including emotions, due to its close association with cognitive processes and its ability to elucidate the basic neural mechanisms underlying emotional experiences. EEG is widely recognized for its excellent temporal resolution, which allows researchers to capture rapid changes in brain activity associated with cognitive and emotional processes. Numerous reviews and meta-analyses have reinforced the effectiveness of EEG in capturing the dynamic interplay between neural mechanisms and emotional states. A systematic review and bibliometric analysis by Ismail and Karwowski examined how EEG indices are used to quantify human cognitive functions and highlighted EEG’s high temporal resolution and sensitivity to various cognitive functions such as fatigue, workload, and stress^13^. In the realm of emotion research, Gkintoni et al. explored EEG-based emotion research from the perspective of integrating neural and emotional networks, and they found that EEG’s capability extends not only to cognitive assessment but also to understanding affective states in neuroscience research^14^. Recent meta-analyses in education and affective computing also highlight EEG’s growing relevance. For example, D’Mello and Kory reviewed various physiological sensing techniques and concluded that EEG is one of the most promising modalities for studying emotions in educational settings^15^. Given this robust evidence, these findings collectively highlight EEG’s strength in capturing the dynamic nature of brain activity, offering researchers and practitioners a powerful tool to explore and understand the neural underpinnings of cognitive functions, including emotions.

Recent advances in EEG signal analysis, particularly in the realm of functional connectivity, have provided novel insights into the brain dynamics underlying cognitive and emotional processes. Investigating EEG functional connectivity allows for a more comprehensive characterization of how these processes are supported by dynamic neural communication. Increasing evidence suggests that distinct emotional and cognitive states, such as boredom, mind wandering, or focused attention, are associated with unique patterns of neural activity and connectivity. For example, during resting-state conditions, functional connectivity is typically dominated by activity in the default mode network (DMN), i.e., a group of brain regions that show higher activity when a person is not engaged in any specific external task^16^. During mind wandering, which often occurs when attention drifts away from the present task, the DMN becomes more active, while brain regions involved in attention and executive control may show reduced connectivity^17^. In contrast, boredom, though also characterized by disengagement from the external environment, may involve a distinct neural signature^18^. Research has shown that boredom is associated with reduced connectivity within the DMN, reflecting a lack of stimulating internal mental activity^16^, as well as increased variability in network switching^16^, which may represent an internal search for cognitive stimulation. These distinctions suggest that different cognitive states vary not only in their experience but also in their underlying network-level coordination.

Literature increasingly supports the notion that various emotional states correspond to unique patterns of neural response and connectivity. For instance, Hossein and Sahar^19^ found that exposure to joyful stimuli leads to greater beta-band coherence connectivity, as indicated by increased nodal strength and enhanced clustering in both inter- and intra-hemispheric networks, relative to neutral or melancholic stimuli. Zhang et al. demonstrated that negative emotions (sadness, fear, and disgust) were associated with increased functional connectivity, specifically, greater coherence and phase synchronization in the beta and gamma bands across distributed electrode sites^20^. Lee et al. utilized connectivity indices such as correlation, coherence, and phase synchronization to compute brain functional connectivity in EEG signals associated with different emotional states^21^. Their findings revealed distinct connectivity patterns across emotional conditions, with notable significant differences observed across various electrode sites. Frequency-specific modulations were observed primarily in the alpha and beta bands, where positive emotions were linked to stronger alpha-band connectivity, and negative emotions were associated with increased beta-band connectivity. Li et al. found notable differences in the theta-band connectivity, assessed using the phase-locking value measure, when comparing negative and neutral emotional stimuli. The results suggest that negative stimuli engage distinct patterns of neural synchronization, indicating the involvement of differentiated functional networks in emotional processing^22^. Kilic and Aydin^23^ demonstrated that graph-theoretical metrics—particularly nodal strength and clustering coefficients were effective in differentiating between discrete emotional states such as fear and happiness using EEG data. Their study involved constructing functional connectivity networks using Pearson and Spearman correlation coefficients between all pairs of EEG channels and computing graph measures to characterize the brain’s topological organization during emotional processing. They found that negative emotions like fear were associated with higher nodal strength and increased clustering, indicating more localized and dense connectivity patterns. In contrast, positive emotions like happiness exhibited lower clustering and more distributed network organization, reflecting broader integration across brain regions. Aydin^24^ examined emotion regulation strategies using direct transfer function connectivity measure and found that cognitive reappraisal was associated with increased global efficiency and reduced modularity, indicating more integrated and cohesive brain network activity. Recently, Aydin and Onbasi^25^ analyzed EEG responses to music stimuli designed to evoke fear and anger, using coherence measures to examine the underlying neural connectivity patterns. Their findings revealed that negative emotions were associated with increased clustering coefficients, suggesting a heightened tendency for localized interactions among brain regions. Simultaneously, they observed reduced global efficiency and longer characteristic path lengths, indicating decreased overall network integration and communication efficiency. All these findings underscore the relevance of brain connectivity patterns that change dynamically in response to different emotional stimuli, providing a more nuanced understanding of the neural mechanisms underlying emotion. However, understanding the differences in brain connectivity during boredom is largely underexplored. By elucidating the patterns of functional connectivity associated with boredom, researchers can gain insight into the network dynamics and neural circuits implicated in the experience of boredom.

The present study aims to investigate EEG functional connectivity associated with boredom, relative to neutral states, to better understand the brain dynamics underlying boredom and its implications for learning. To achieve this, an educational lecture video was designed to passively induce boredom in participants while their EEG data could be collected. Using a coherence method, we construct functional connectivity matrices across various EEG frequency bands, measuring the degree of synchronization between different brain regions. Several functional connectivity measures have been utilized in the literature, we employed spectral coherence in this study due to its ability to effectively capture frequency-specific synchronization between EEG signals^26^. Spectral coherence is particularly well-suited for analyzing oscillatory brain activity related to emotional processing^27^, offering a balance of interpretability and computational efficiency. Its widespread use in prior emotion recognition research further supports its suitability for capturing meaningful connectivity patterns within specific frequency bands. We hypothesize that boredom will be associated with altered coherence connectivity patterns, potentially characterized by increased connectivity among brain regions that are typically active during states of rest, mind-wandering, or self-referential thinking. By examining connectivity dynamics, we expect to uncover the specific neural mechanisms that underline the experience of boredom, thereby contributing valuable insights to educational psychology and neuroscience. To the best of the authors’ knowledge, the present study is the first to explore the differences in functional brain networks during boredom in healthy adults using EEG.

## Materials

### Participants

Eighty-four university students (38 males and 46 females) aged between 21 and 45 years (mean age = 26.90 ± 5.29 years) participated in this study, following recruitment in Nanyang Technological University, Singapore. All participants were briefed on the study and provided informed consent prior to participation in accordance with the Declaration of Helsinki. Participants were all right-handed, reported normal or corrected-to-normal vision, and had no history of neurological injuries (e.g., stroke), neurological disorders (e.g., epilepsy), or head trauma. Participants were evenly distributed in terms of gender (54.7% female and 45.3% male), with a chi-squared test indicating no significant difference in gender distribution, χ2(1) = 0.762, *p* = 0.383. An independent samples t-test was conducted to assess whether there was a significant difference in age between male and female participants. The analysis showed no significant difference in mean age between males and females (mean ± SD: males = 27.0 ± 4.87 years; females = 26.82 ± 5.66 years; t (82) = 0.16, *p* = 0.88), indicating that the groups matched in age.

### Ethical statement

The study procedures were approved by the Nanyang Technological University (NTU) Institutional Review Board (IRB-2022-1048). After completion, participants were compensated with 10 Singapore Dollars for their time.

### Boredom stimulus and experiment protocol

**Fig. 1:**
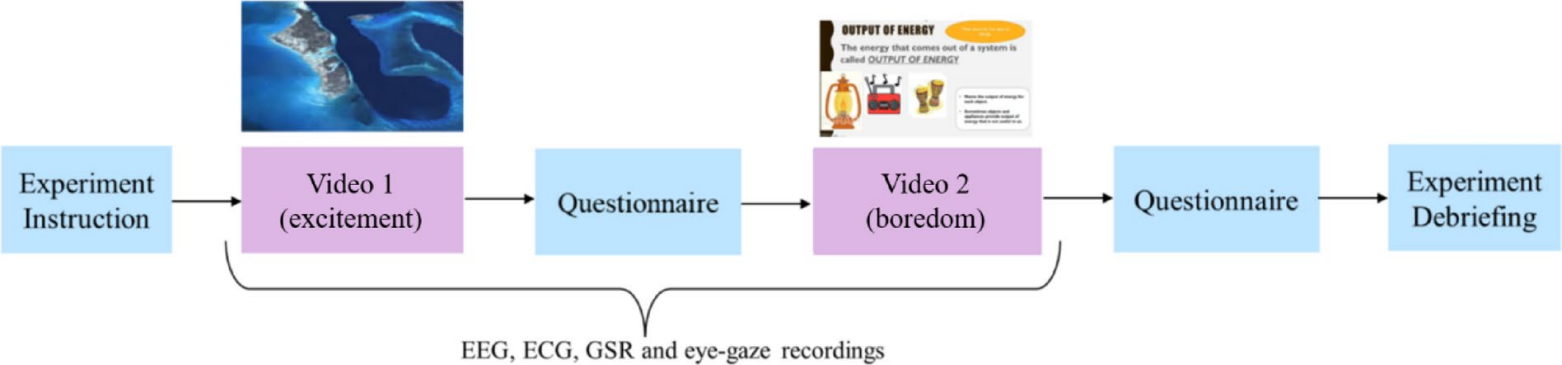
The boredom induction protocol.

Figure 1 illustrates the boredom induction protocol designed for this study. The experimental protocol comprised EEG recordings and subjective self-report measures of emotion in response to two videos: (i) a non-boring video, and (ii) a boring educational video. Both videos were presented to participants using E-Prime software (Version 3.0; Psychology Software Tools; https://pstnet.com/products/e-prime/) and displayed on a 345 mm × 195 mm laptop monitor with a resolution of 1024 × 768 pixels at 60.04 Hz. The total duration of the experiment was approximately 60 min.

Each participant viewed two video stimuli that elicit different affective states: neutral and boredom. To set a baseline state of neutral, participants first watched a 4.33-minute video taken from the British Broadcasting Company’s (BBC) documentary, “Planet Earth”, showing colorful scenes of marine life accompanied by narration and music^28^. This video was selected based on its prior validation as a neutral stimulus in the study by Merrifield and Danckert^29^, where it was shown to elicit minimal affective responses across participants. However, this study’s video increased self-reported excitement rather than inducing a neutral affective state (see Table 1). Nevertheless, we reasoned that excitement would be helpful to examine alongside boredom. As such, the “neutral” video will henceforth be referred to as the “excitement” video.

After the baseline video, a boring 10-minute-long educational video was shown to the participants, involving a science teacher explaining input/output energy concepts in standard lecture format. This lecturer’s video was selected after a pilot was conducted to ensure the video reliably induced a strong feeling of boredom. We also ensured that the selected videos had common boredom antecedents, such as a lack of cognitive stimulation (i.e., challenge or cognitive demand) and the use of slow, monotonous voices in the video featuring low-value content^5,30^. Moreover, viewing video-recorded lectures is a standard and important learning format, particularly for schools utilizing blended and online learning. In addition, this passive video paradigm is considered advantageous for boredom research in education contexts as the simplicity of the task enables the isolation and assessment of boredom with minimal influence from other factors that may be present in active behavioural or learning tasks. During the experiment, the exciting video was presented before the boring video. This fixed order was chosen to neutralize the participants’ emotional states with the task before inducing boredom. The study involved only one trial per condition, so randomization or counterbalancing was not implemented to avoid carry-over effects and unnecessary variability.

Participants were allowed to stop watching the boredom video after a minimum of 6 min and move on to the next stage. This was to maximize data recording while ensuring a minimum of 6 min of EEG data related to a boring video stimulus. Thus, the play time of each participant was different. In our analysis, 46.43% of students stopped watching the boring video early, and the average play time was 8.50 ± 1.10 min. Immediately after each video stimulus, participants were given a 90-second interval-stimulus interval to complete a paper-based State Affect Questionnaire (SAQ) (see Appendix 1), adapted from the Positive and Negative Affect Scale (PANAS)^31^. Merrifield and Danckert^29^ previously used an adapted version of the SAQ, derived from PANAS, to assess emotional states, including boredom, in experimental settings. Their version included 24 emotion terms and demonstrated sensitivity to affective changes induced by video stimuli. In our study, we similarly adapted the SAQ to capture transient affective states relevant to the experimental context, which consisted of five emotions (excitement, disgust, neutral, boredom, and distress). The primary purpose of the SAQ was to measure the participants’ momentary emotional responses to the stimuli and to confirm the existence of boredom in the presented video. Although standardized boredom scales such as the Multidimensional State Boredom Scale (MSBS)^2^ and the Boredom Proneness Scale^32^ (BPS) offer comprehensive assessments, they are generally designed for trait-level or longer-form assessments. Given our focus on assessing immediate affective responses following stimulus exposure within a controlled, time-limited experimental design, these existing measures were not feasible for our study. Instead, the SAQ was chosen to efficiently capture transient affective states within a short task window, minimizing participant burden. The participants indicated what emotions they felt and the intensity of those emotions on the scale of 0 (none) to 8 (extreme).

### Scalp EEG recordings

In this research, continuous scalp EEG, electrocardiogram, and eye-gaze data were simultaneously collected at 1000 Hz using an AntNeuro EEGO sports amplifier. Additionally, galvanic skin response data were collected using Shimmer at 128 Hz. For this study, only EEG data were obtained and analyzed. Synchronization between the EEG data and video stimuli was achieved using a customized E-Prime script in conjunction with Psychology Software Tools Chronos. Chronos was used to send hardware-based event markers to the AntNeuro EEGO sports amplifier via parallel port connection, marking the onset and offset of excitement and boredom video clips. This ensured precise temporal alignment between stimulus presentation and EEG recordings. The raw scalp EEG signals were collected from a 64-channel gel electrode cap (Fp1, Fpz, Fp2, F7, F3, Fz, F4, F8, FC5, FC1, FC2, FC6, T7, C3, Cz, C4, T8, CP5, CP1, CP2, CP6, P7, P3, Pz, P4, P8, POz, O1, O2, AF7, AF3, AF4, AF8, F5, F1, F2, F6, FC3, FCz, FC4, C5, C1, C2, C6, CP3, CP4, P5, P1, P2, P6, PO5, PO3, PO4, PO6, FT7, FT8, TP7, TP8, PO7, PO8, and Oz), including the left mastoid (M1), right mastoid (M2), and electrooculogram (EOG). The electrodes were positioned according to the international standard 10–10 electrode system, grounded at AFz, with CPz as the active reference. Electrode impedance was set below 10 KΩ during electrode placement.

### Procedure

**Fig. 2:**
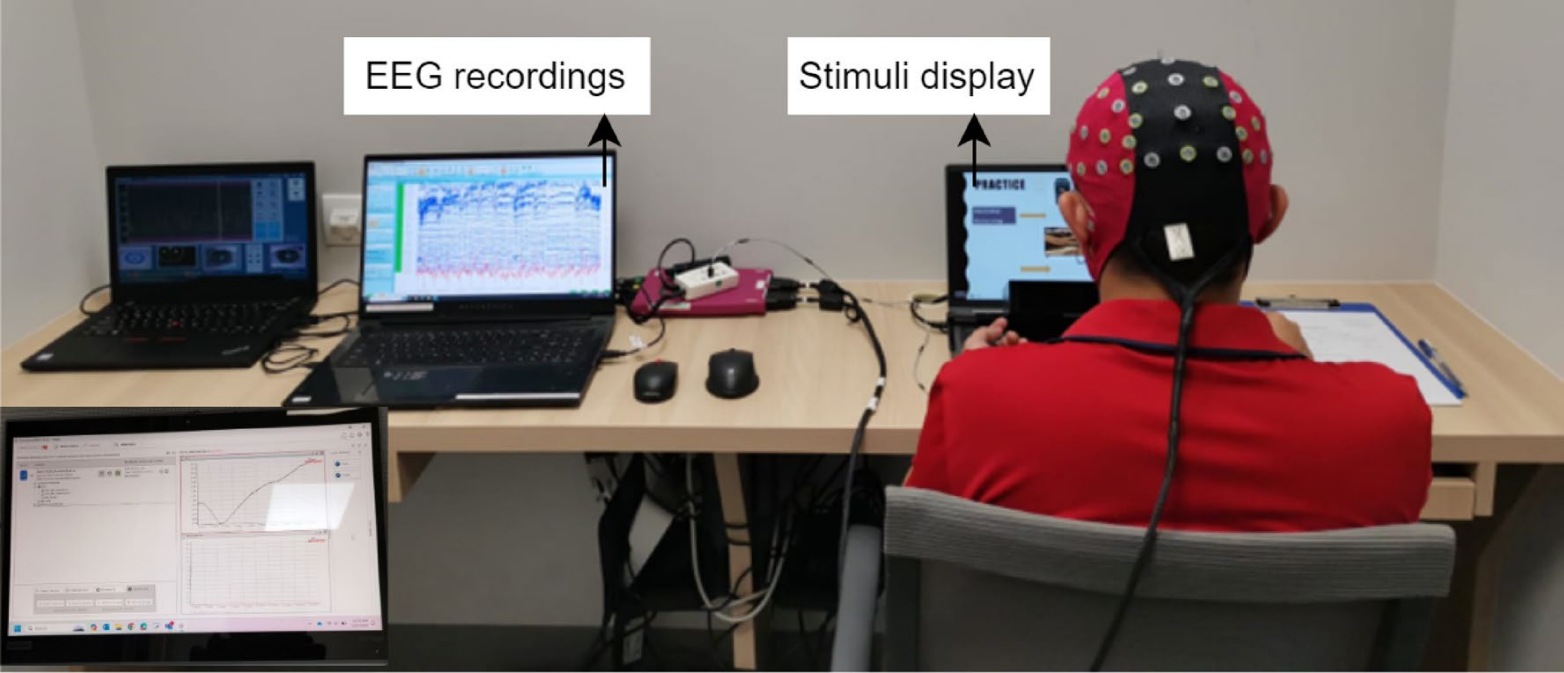
Experimental setup at the SoLEC lab, NIE.

**Fig. 3:**
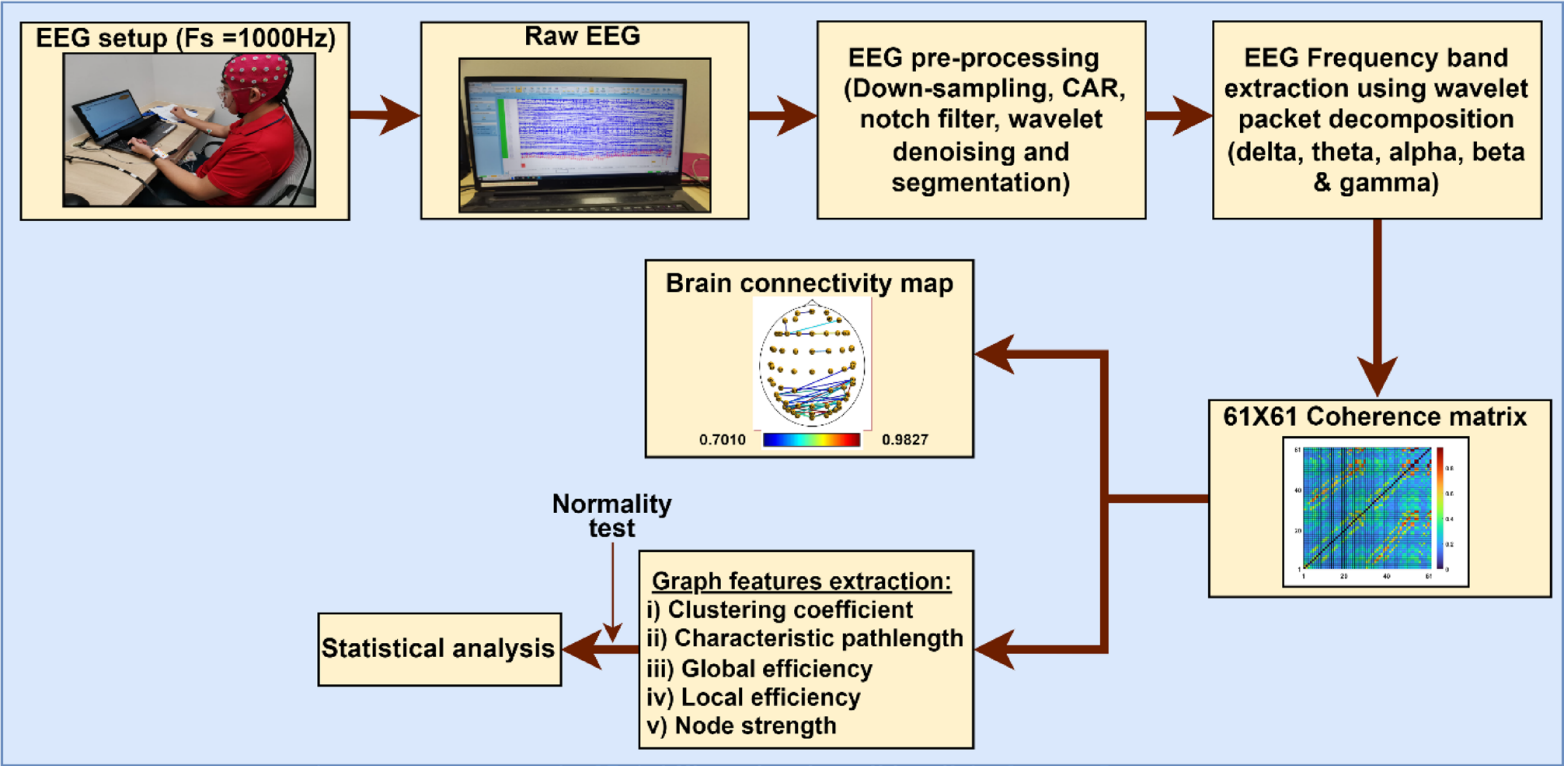
A framework for an entire method employed in this investigation.

Figure 2 shows the complete experimental setup for this study. The experiment took place in a quiet environment at the Science of Learning Education Centre (SoLEC) at the National Institute of Education (NIE), NTU. Participants attended the sessions individually. Upon arrival, participants were briefed on the procedure and asked for their informed consent to complete the experiment. During the briefing, the purpose of the experiment (i.e., to explore boredom) was not revealed to avoid affecting the participants’ behaviour. Participants were seated comfortably and equipped with EEG sensors to watch the videos. Following the completion of the experimental session, we debriefed the participants, explaining the intent and goals of the study. Approval and informed consent for publishing the person’s image in an online open-access journal have been obtained for Figs. 2 and 3.

## Methods

### EEG data annotation

Participant-reported data from the SAQ served as the ground truth for labeling emotional states corresponding to each video. Initially, EEG recordings were collected from 84 individuals as they viewed excitement and boredom stimuli. Based on their SAQ responses indicating the emotions they experienced while watching each video stimulus, each participant’s EEG recording was annotated to reflect their experienced emotional state. Eleven participants reported not feeling bored while watching the science lecture video (i.e., the boredom stimulus) or feeling bored while watching the Planet Earth video (i.e., the excitement stimulus) and were excluded from further analysis. As a result, the final dataset comprised EEG data from 73 participants.

Figure 3 shows the entire method employed in this investigation. After the data annotation, the raw EEG data were subjected to (1) preprocessing, (2) functional connectivity matrix construction, (3) network measures computation, and (4) statistical analysis and visualization of brain connectivity.

To implement the methods and analyze the results described in the subsequent sections, MATLAB 2023b was utilized for EEG preprocessing, segmentation, decomposition, matrix construction, and network measures computation. The MATLAB scripts and statistics tests were executed on an HP laptop running a 64-bit Windows operating system, equipped with a 12th Gen Intel^®^ Core™ i7- 12,800 H processor at 2.40 GHz and 32 GB of RAM.

### EEG signal preprocessing

Raw continuous scalp EEG data were first down-sampled to 256 Hz to reduce computational complexity, re-referenced to the common average, and notch-filtered at 50 Hz to eliminate line noise. A threshold-based wavelet denoising method was then applied to remove the EOG artifact. Daubechies wavelet (db9) with level 6 was chosen as the mother wavelet, and Stein’s Unbiased Risk Estimate (SURE) thresholding algorithm was used^33^. Wavelet denoising was selected due to its ability to handle non-stationary and overlapping artifacts without requiring long continuous data segments or manual component selection^34^. This makes it particularly suitable for short, task-related EEG recordings, where preserving both temporal and spectral characteristics is crucial. Unlike conventional techniques such as Independent Component Analysis (ICA), which assumes statistical independence, or Artifact Subspace Reconstruction (ASR), which relies on clean calibration data, wavelet denoising offers a more adaptive and data-driven approach to artifact removal^35,36^. The exciting stimulus video EEG data and the last 4.2 min of the boring video EEG data were then obtained for further analysis; this latter step was to match the neutral video recording length and isolate the period where boredom is likely to be highest. Following this, the preprocessed EEG from each video condition was then segmented into 2-second non-overlapping epochs^37^, which allows for capturing the dynamic fluctuations in brain connectivity over time. The epoched data were then decomposed into five standard EEG sub-frequency bands namely, delta (0–4 Hz), theta (4–8 Hz), alpha (8–16 Hz), beta (16–32 Hz) and gamma (32–64 Hz) across all EEG channels using the wavelet packet transform, using a Daubechies wavelet of db9 and decomposition level 6. Compared to traditional band-pass filtering, wavelet-based decomposition was chosen for its ability to provide both time and frequency localization, making it well-suited for analyzing the non-stationary and transient nature of EEG signals^38^. Additionally, it enables efficient separation of overlapping frequency components without introducing phase distortion, making it suitable for task-related EEG data such as emotional responses^39^.

### Coherence and construction of EEG functional connectivity matrices

In network theory, nodes and edges form the fundamental components of connectivity between nodes. This study considers each EEG electrode a node, and the connection strength between nodes is represented as an edge. Spectral coherence was measured for functional connectivity analysis due to its ability to capture both phase and amplitude relationships between oscillatory signal data, providing a more nuanced understanding of brain interactions^40^. Additionally, spectral coherence is robust to noise and allows for frequency-specific assessments, making it a reliable method for investigating dynamic connectivity patterns in EEG^41^, specifically in emotion research^27,42^. Furthermore, coherence features and cognitive measures offer validation of diagnostic problems and material to guide and choose targeted interventions^43,44^.

**Fig. 4:**
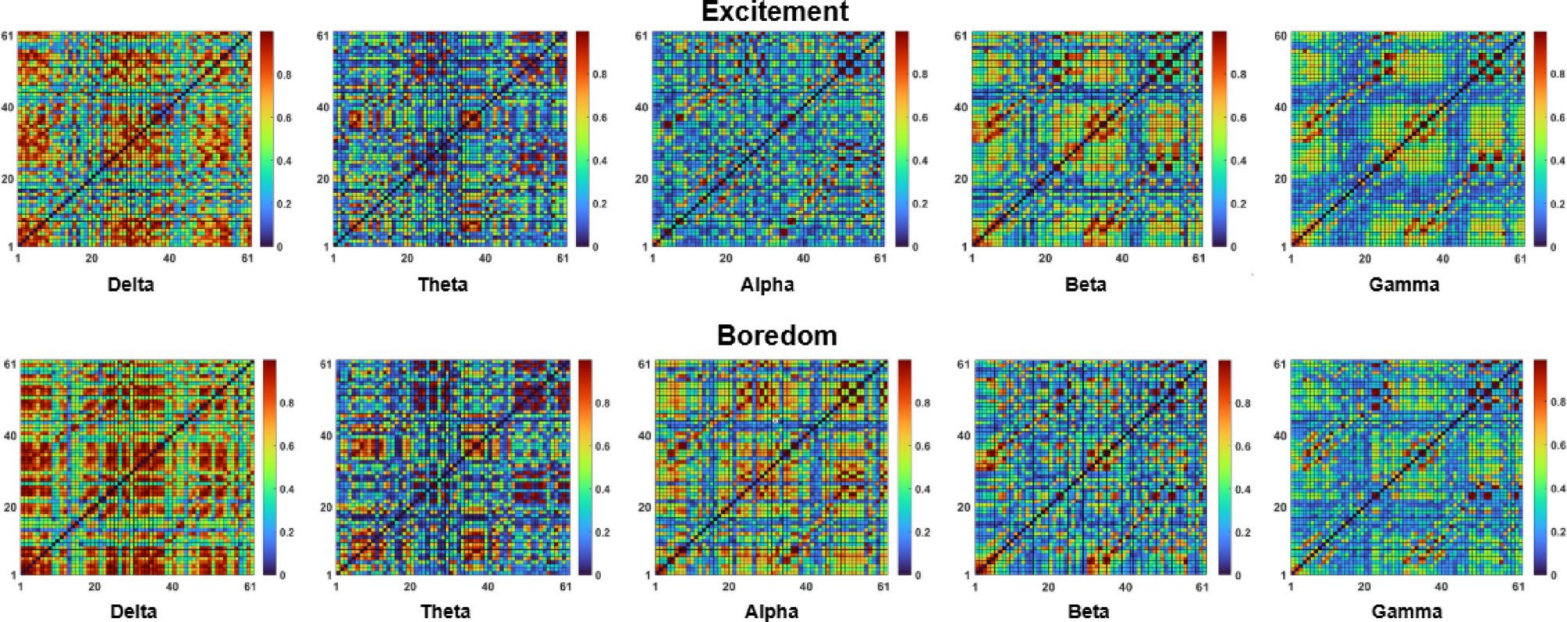
Visualization of connectivity matrices (without thresholding) computed using coherence from a 2-second EEG epoch of the decomposed EEG signals from a sample participant. One representative matrix was selected to illustrate the network structure under both boredom and excitement. The matrices may appear overly dense because coherence values were calculated for all possible channel pairs, resulting in a fully connected matrix with varying connection strengths. The first row shows examples from excitement, and the second row shows examples from boredom state for EEG frequency bands delta (0–4 Hz), theta (4–8 Hz), alpha (8–16 Hz), beta (16–32 Hz), and gamma (32–64 Hz), respectively. The connectivity matrix x-axis and y-axis denote the EEG channel number.

Coherence $\:{(C}_{xy})$ of signal $\:x$ is defined as the measure of the degree of association between two signals $\:x\left(t\right)$ and $\:y\left(t\right)$ in the frequency domain, as described in Eq. (1), with values ranges between [0,1], where the value closer to 1 represents a stronger correlation between the two signals.

1 $$\:{C}_{xy\:}\left(f\right)=\:\frac{{\left|{P}_{xy}\left(f\right)\right|}^{2}}{\left|{P}_{xx}\left(f\right)\right|\left|{P}_{yy}\left(f\right)\right|}$$

2 $$\:{C}_{xy}=\:{\left[\begin{array}{c}{\varvec{C}}_{11}\:\:\:{C}_{12}\:\:\:{C}_{13}\:\:\dots\:.\:\:{C}_{1n}\\\:{C}_{21}\:\:\:{\varvec{C}}_{22}\:\:\:{C}_{23}\:\:\dots\:.\:\:{C}_{2n}\\\:{C}_{31}\:\:\:{C}_{32}\:\:\:{\varvec{C}}_{33}\:\:\dots\:.\:\:{C}_{3n}\\\:\:\:\::\:\:\:\:\:\:\:\:\::\:\:\:\:\:\:\:\:\:\::\:\:\:\dots\:.\:\:\:\:\:\:\::\\\:{C}_{n1}\:\:\:{C}_{n2}\:\:\:{C}_{n3}\:\:\dots\:.\:\:{\varvec{C}}_{\varvec{n}\varvec{n}}\end{array}\right]}_{\:\:61\times\:61}$$

where $\:{P}_{xy}\:$denote the cross power spectral density (CPSD) of $\:x\left(t\right)$ and $\:y\left(t\right)$; $\:{P}_{xx}$ is the PSD of $\:x\left(t\right)$.

and $\:{P}_{yy}$ is the PSD of $\:y\left(t\right)$. In this study, 61 channels were utilized, resulting in a 61 × 61 undirected weighted coherence matrix $\:\left({C}_{xy}\right)\:$for each 2-second epoch of the decomposed EEG signals across different frequency bands (see Eq. (2)). This matrix was neither thresholded nor binarized; instead, the full weighted matrix was retained and served as the functional connectivity matrix for subsequent network measure computation. By preserving the coherence values between all pairs of EEG channels, this approach allows us to maintain fine-grained information about the strength and distribution of functional interactions across the brain. Moreover, using weighted matrix allowed for a more realistic representation of brain dynamics, avoiding the arbitrary selection of threshold values, which can significantly influence network topology and introduce bias. Each value in the matrix represents the coherence value between pairs of channels. Figure 4 presents connectivity matrices computed using coherence for boredom and excitement states across each frequency band.

### EEG functional connectivity network analysis

Five network measures, namely clustering coefficient $\:\left({C}_{eff}\right)$, characteristic path length $\:\left({C}_{pl}\right)$, global efficiency $\:\left({E}_{glo}\right)$, local efficiency $\:\left({E}_{loc}\right)$, and node strength $\:\left({N}_{str}\right)$were calculated independently for excitement and boredom states from each 2-second connectivity matrix. All these network measures were calculated using the brain connectivity toolbox (BCT)^40^ in MATLAB.

*Clustering coefficient* $\:\left({C}_{eff}\right)$: The clustering coefficient is a measure of network segregation, calculated as the ratio of existing connections among a node’s nearest neighbors to the maximum possible connections between them. A high $\:{C}_{eff}$ indicates that nodes tend to form densely connected local clusters, suggesting efficient local information transfer^40,41^. The mathematical formula for the $\:{C}_{i}$ is the following Eq. (3):

3 $$\:{C}_{eff}=\frac{1}{N}{\sum\:}_{i=1}^{N}\frac{{E}_{i}}{{D}_{i}\left({D}_{i}-1\right)/2}$$

Here $\:N$ denotes the number of nodes in the network $\:Z$ ($\:Z=61$ in this work), $\:{E}_{i}$ is the number of edges between neighbors of node $\:i$, and $\:{D}_{i}$ denotes the number of neighbors of node $\:i$.

*Characteristic pathlength* $\:\left({C}_{pl}\right)$: The characteristic path length quantifies the average shortest path between all pairs of nodes in a network, as calculated in Eq. (4). It indicates the efficiency of information transfer across the network. A low $\:{C}_{pl}$ ensures rapid transmission of information in the whole network^40,41^.

4 $$\:{C}_{pl}=\frac{1}{N(N-1)}{\sum\:}_{i=1,i\ne\:j\in\:Z}^{N}{L}_{ij}$$

where $\:{L}_{ij}$ is the shortest path length between nodes $\:i$ and $\:j$.

*Global efficiency* $\:\left({E}_{glo}\right)$: Global efficiency is a method to measure the overall efficiency of information transfer across a network and calculated as mentioned in Eq. (5). It quantifies how well information can be communicated throughout the entire network by averaging the inverse of the shortest path lengths between all pairs of nodes^40^. In the network analysis, higher global efficiency indicates that signals can be transmitted more effectively between different brain regions, reflecting a well-integrated and functionally connected neural system^41^.

5 $$\:{E}_{glo}=\frac{1}{N(N-1)}\sum\:_{i\ne\:j\in\:Z}\frac{1}{{L}_{ij}}$$

*Local efficiency* $\:\left({E}_{loc}\right)$: The $\:{E}_{loc}$ measures the efficiency of information transfer among the immediate neighbors of a given node within the network. In the context of brain networks, higher local efficiency indicates robust local communication and integration among neighboring brain regions^40,41^. The $\:{E}_{loc}$ of a network was calculated as in Eq. (6)

6 $$\:{E}_{loc}=\frac{1}{N}\sum\:_{i\in\:Z}{E}_{loc}\left(i\right)$$

where $\:{E}_{loc}\left(i\right)$ is the local efficiency of node $\:i$.

*Node strength* $\:\left({N}_{str}\right)$: The node strength $\:{N}_{str}$defines the overall connectivity of a specific node within a network. It is defined as the sum of the weights of the edges connected to that node^40^. Mathematically, for a node $\:i$, the node strength $\:{N}_{str\:\left(i\right)}$ can be expressed as Eq. (7):

7 $$\:{N}_{str\:\left(i\right)}=\sum\:_{jϵN\left(i\right)}{w}_{ij}$$

where $\:{N}_{str\:\left(i\right)}$ is the strength of the node $\:i;$ $\:{w}_{ij}\:$is the weight of the edge connecting the node $\:i$ to node $\:j$; $\:N\left(i\right)$ is the set of neighbors connected to the node $\:i$.

In addition, brain functional connectivity was visualized using BrainNet Viewer, a MATLAB-based brain network visualization toolbox^45^. The 61 × 61 connectivity matrices derived from coherence values were input into the BrainNet viewer to visualize the brain networks in excitement and boredom states. This results in the graphical representation of connectivity patterns by mapping network nodes and edges to visualize spatial and temporal networks of the brain in a clear and visually informative manner. The matrices were thresholded to 0.6 when generating the connectivity plot to visualize only the strong and moderate connections^40^. A threshold of 0.6 is commonly used in EEG coherence analyses to exclude weak or potentially spurious connections, thereby highlighting more meaningful patterns of network organization. Only edges (i.e., connections between EEG channels) with coherence values greater than 0.6 were retained and displayed. Connections with coherence values equal to or below 0.6 were omitted to enhance the visibility of moderate to strong functional connectivity patterns. Furthermore, brain topoplots were generated using a customized MATLAB script to visualize significant brain regions. To achieve this, z-score transformation was performed for each condition (boredom vs. excited state) across all EEG channels. Node strength values for the boredom and the excited state were compared, and the resulting z-scores were used to create the brain topoplots, allowing clear visualization of regions with significant differences. It is important to note that the z-score transformation was applied solely for the purpose of visualizing brain regions with prominent node strengths in each state.

### Statistical analysis

Network features were analyzed using IBM SPSS Statistics for Windows, Version 23.0 (IBM Corp). All network measures were tested for normality. A non-parametric approach was employed for statistical analysis, as the network parameters did not meet the normality assumptions based on the Shapiro-Wilk and Kolmogorov-Smirnov tests. The Wilcoxon signed rank test was employed as the non-parametric test to compare the network measures between the excitement and boredom states. The alpha level was set at 0.05 for all statistical analyses. $\:{C}_{eff}$, $\:{C}_{pl},\:{E}_{glo},\:{E}_{loc}$, and $\:{N}_{str}\:$were considered as dependent variables at different EEG bands, whereas the excitement and boredom states were considered as independent variables. Bonferroni correction was applied to adjust the significance level for multiple comparisons. The new *p*-value was set to 0.002 (0.05/5*4 (EEG bands*features)) when comparing the $\:{C}_{eff}$, $\:{C}_{pl},\:{E}_{glo},\:$and $\:{E}_{loc}$. For $\:{N}_{str}$, the new p-value was set to 0.0001 (0.05/61 (channels)*5 (bands)). The SAQ ratings were analyzed using separate two-way repeated measures ANOVA (analysis of variance), with video type (excitement, and boredom) and emotion (excitement, disgust, neutral, boredom, and distress) as within-subject factors, to examine their main effects and interaction on the SAQ scores. Significant main effects and interactions were further explored using post hoc pairwise comparisons with Bonferroni correction.

## Results

### Boredom functional connectivity

Table 1 presents the averages and standard deviations (SD) of all participants’ self-reported ratings on SAQ for excitement and boredom stimuli. The two-way ANOVA analysis revealed a significant main effect of video type, F (1, 72) = 11.576, *p* < 0.001, and a significant main effect of emotion, F (4, 69) = 11.983, *p* < 0.001. Additionally, there was a significant interaction between video type and emotion, F (4, 69) = 60.499, *p* < 0.001. Post hoc comparisons indicated that SAQ ratings for the boredom video (mean ± SD = 5.28 ± 2.67) were significantly higher (*p* < 0.001), relative to all other emotions, supporting the validity of the stimulus for boredom induction. For video 1, most of the participants significantly endorsed feelings of excitement (mean ± SD = 2.97 ± 2.77, *p* < 0.001) or neutral emotion (mean ± SD = 2.202 ± 2.54, *p* < 0.001), with very few indicating that they felt bored. Thus, while not purely neutral, the BBC video may still be considered an apt comparison for boredom.

**Table 1:** Averages ± SD of all participants’ self-report ratings on SAQ. Bolded items represent the strongly endorsed emotion.

S. No	Video	Emotion	Average intensity
1	Excitement	Excitement	**2.976 ± 2.77**
		Disgust	0.190 ± 0.85
		Neutral	2.202 ± 2.54
		Boredom	0.773 ± 1.82
		Distress	0.130 ± 0.87
2	Boredom	Excitement	0.380 ± 1.19
		Disgust	0.250 ± 1.19
		Neutral	0.916 ± 1.98
		Boredom	**5.285 ± 2.67**
		Distress	0.738 ± 1.84

**Fig. 5:**
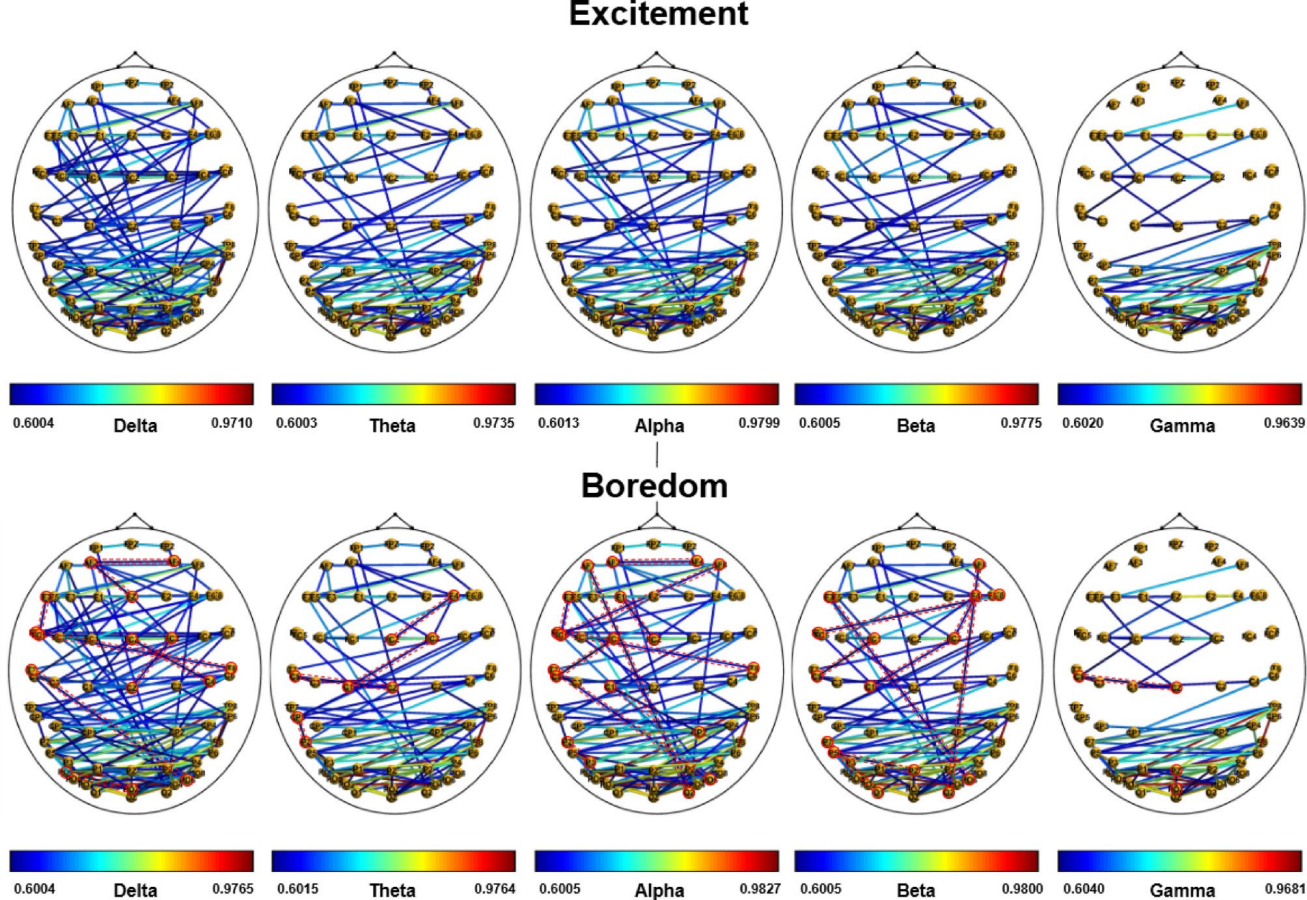
Functional connectivity maps for the excitement (top) and boredom (bottom) state (for visualization purposes, matrices are threshold at 0.6 only to show stronger connections). The red dotted lines show the new connections in the boredom state.

Figure 5 shows brain connectivity maps for excitement and boredom processing in different EEG frequency bands (delta, theta, alpha, beta, and gamma). Only moderate and strong connections (above 0.6) are displayed. In the delta band, there is no significant difference in connectivity between excitement and boredom emotional states, except for a few connections (AF3-AF4, FCz-C4, F7-FC5, AF3-Fz, FC2-Cz, T7-PO6, T8-FC7, and POz-PO7) that appear only during boredom. In the theta band, all major brain regions—occipital, parietal, temporal, and frontal—are involved in processing excitement and boredom emotional states. For boredom, new connections appear between FC2-C1, F4-FCz, C5-Cz, and CP5-P7, while the connection at F6-FC5 disappears. In the alpha band, similar regions are involved for both states. During boredom, additional connections (AF3-AF4, F7-FC5, FC1-C5, AF8-FC5, F1-FCz, T8-FC7, T7-PO6, C1-CPz, P7-P3, and AF7-O2) are observed, which are absent in the exciting state processing. In the beta band, new connections (F7-PO6, F4-FC2, FC7-F8, FC1-C5, AF8-O2, C1-FC2, P5-P2, and P7-O1) appear in boredom processing that are not present in the exciting state, while CP5-C6 and C2-CP6 are disconnected. In the gamma band, connectivity patterns differ from other bands, with no prefrontal and frontal regions involvement. However, occipital and parietal regions are still active in both states. A new connection (C5-Cz and Pz-POz) appears during boredom, while CP3-C4 is disconnected. Across all bands, moderate to strong connections are observed between the occipital, parietal, temporal, and frontal regions, highlighting the importance of these areas in processing emotions.

**Fig. 6:**
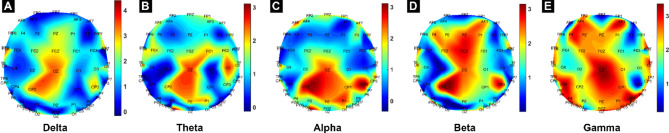
Statistical topography of node strength comparison between excitement and boredom states for each EEG frequency band. (A) delta, (B) theta, (C) alpha, (D) beta, and (E) gamma. The color bar represents the *z*-values of the electrode node strength when comparing excitement vs. boredom emotional state.

Figure 6 illustrates the key topographic areas exhibiting significant z-values when comparing node strengths between boredom and excitement stimuli. Notably, the gamma band coherence revealed larger and more notable differences in brain node strengths compared to other EEG bands, indicating its effectiveness in distinguishing between boredom and excitement emotion states. The beta band coherence also showed considerable differences in node strengths, particularly in the frontal and central regions. In contrast, the delta, theta, and alpha bands demonstrated observable node strength differences primarily in the central regions. The topographical analysis further highlights that higher frequency EEG bands, such as gamma and beta, show more pronounced activation patterns during the processing of boredom compared to excitement stimuli.

### Network measures in excitement and boredom stimulus

**Fig. 7:**
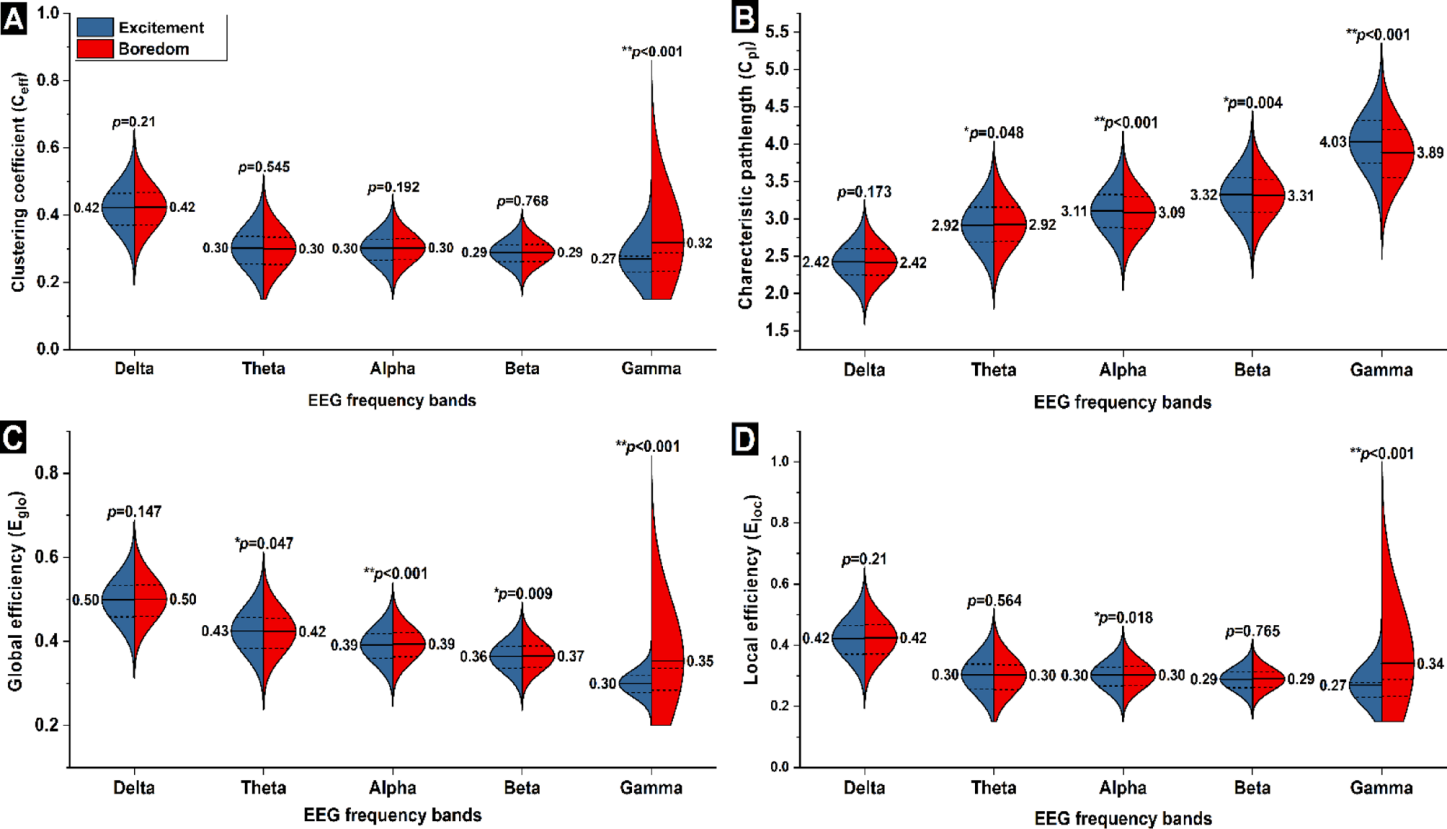
Comparison of network measures between excitement and boredom state for delta, theta, alpha, beta, and gamma frequency bands coherence using a split violin plot. (A) clustering coefficient, (B) characteristic path length, (C) global efficiency, and (D) local efficiency. The p-value of each frequency band of excitement vs. boredom is depicted for each measure. In the violin plot, the solid line represents the group’s mean. *p < 0.05, and **p_corrected_<0.002.

Figure 7 shows the distribution of $\:{C}_{eff},$ $\:{C}_{pl}$, $\:{E}_{glo}$, and $\:{E}_{loc}$values of excitement and boredom using a split violin plot. In the violin plot, the solid black line denotes the group mean, while the dotted line denotes the squared error. When comparing the $\:{C}_{eff}$ (Fig. 7A) coherence values, we found a substantial significant difference in the gamma band coherence (z = -14.79, *p* < 0.001). The mean $\:{C}_{eff}$ was considerably higher in the boredom state compared to the excitement state. When comparing the $\:{C}_{pl}$ coherence values between two states (Fig. 7B), significant differences were seen in alpha (z = -4.853, *p* < 0.001), beta (z = -3.186, *p* < 0.05), and gamma (z = -25.36, *p* < 0.001) $\:{C}_{pl}$. During boredom, the mean $\:{C}_{pl}$ was found to be considerably lower than the excitement state in the alpha and beta $\:{C}_{pl}$. We also observed that the mean $\:{C}_{pl}$ was substantially lower in the gamma band $\:{C}_{pl}$. When comparing the $\:{E}_{glo}$ coherence values, significant differences in the $\:{E}_{glo}$ of alpha (z = -4.405, *p* < 0.001), beta (z = -2.605, *p* < 0.05), and gamma (z = -24.17, *p* < 0.001) bands were found. The boredom state $\:{E}_{glo}\:$mean was considerably higher in the alpha and beta bands coherence; the mean $\:{E}_{glo}$ of gamma band coherence was found to be substantially higher. When comparing the $\:{E}_{loc}$ coherence, significant differences in alpha (z = -2.368, *p* < 0.05) and gamma (z = -21.937, *p* < 0.001) bands were found. When comparing excitement to boredom, the $\:{E}_{loc}$ coherence value in the alpha band was found to be lower, whereas the boredom state had much higher $\:{E}_{loc}$ values in the gamma band coherence than the excitement state. Overall, the gamma band coherence showed significant (*p* < 0.001) differences across all the network measures.

## Discussion

The present study analyzed EEG functional brain connectivity associated with boredom while participants watched a boring lecture video. The study results revealed that gamma band coherence exhibited the most notable differences across all the network measures ($\:{C}_{eff},$ $\:{C}_{pl}$, $\:{E}_{glo}$, and $\:{E}_{loc}$) when compared to other frequency bands coherence, such as alpha and beta (see Fig. 7). Notably, gamma band connectivity exhibited distinct patterns during boredom, as reflected by significant changes across several network measures. Specifically, during boredom stimulus, there was an increase in $\:{C}_{eff}$ and $\:{E}_{loc}$, along with alterations in $\:{C}_{pl}$ and $\:{E}_{glo}$, suggesting more locally integrated yet globally less efficient network organization in the gamma frequency range. These findings are consistent with Laufs et al., who reported elevated gamma activity during low-attention or disengaged states^46^. Prior research has also revealed that gamma-band activity supports the transmission of emotional information during the processing of affective stimuli^22,47^. Hence, the observed modulation in gamma-related network metrics may reflect the brain’s shift toward internally directed attention or mind-wandering, commonly associated with emotional disengagement. Furthermore, studies^47–49^ have shown that increased gamma connectivity is often associated with lower levels of cognitive engagement. When individuals are not actively processing external information, their brain networks, particularly in the gamma band, tend to exhibit greater localized coherence and integration, potentially indicating a withdrawal from task-related cognitive processing. This network behavior aligns with the subjective experience of boredom, which is marked by reduced external engagement and diminished cognitive stimulation^50,51^.

This study found minor associations between boredom and network connectivity in theta, alpha and beta frequency bands. In particular, boredom related changes were observed in $\:{C}_{pl}$ and $\:{E}_{glo}$ in the theta band; $\:{C}_{pl}$, $\:{E}_{glo},$ and $\:{E}_{loc}\:$in the alpha band; and $\:{C}_{pl}$ and $\:{E}_{glo}$ in the beta band. The observed alterations in theta-band network measures may reflect a reduction in alertness or an increase in mind-wandering, which often accompanies task disengagement. In the alpha band, slight shifts in connectivity metrics may indicate a transition toward internally focused attention and reduced sensory processing, which are typical of boredom states. Similarly, changes in beta-band network properties could signal diminished cognitive control or weakened top-down attentional modulation during low-engagement conditions^52^. In contrast, no significant associations were found between boredom and connectivity in the delta band. This finding aligns with previous studies suggesting that delta oscillations are more commonly associated with sleep, drowsiness, or pathological states, rather than with the subtle fluctuations in attentional and emotional engagement characteristic of boredom during wakefulness^53^.

The increased gamma band node strength observed in our study (see Fig. 6E) during boredom may reflect the engagement of neural processes involved in maintaining attention and cognitive control in response to a low-stimulation environment, such as watching a monotonous educational video. Gamma band activity is commonly linked to higher-order cognitive functions, including attention regulation, working memory, and the integration of multisensory information^54^. The significantly elevated node strength in the frontal, parietal, temporal, and occipital regions suggests enhanced neural communication and functional integration across these key cortical areas during boredom. This finding aligns with prior research showing that gamma activity plays a central role in coordinating distributed neural processing during cognitively demanding tasks—such as decision-making, sustained attention, and perceptual binding. The observed increase in gamma node strength may represent the brain’s compensatory attempt to remain cognitively engaged, despite the stimulus’s emotionally neutral and repetitive nature. In this context, heightened gamma connectivity could indicate increased internal effort to stay focused or resist disengagement during a boredom-inducing task^55^.

We also observed significantly higher beta band node strength (see Fig. 6D) in the frontal, parietal, and central regions, suggesting enhanced regional connectivity during boredom. This finding is consistent with previous research indicating that beta oscillations are sensitive to subtle fluctuations in emotional and cognitive states, especially in contexts requiring minimal external stimulus engagement^56^. The elevated beta node strength may reflect a compensatory mechanism, whereby the brain increases effort to remain alert and task-focused without stimulating input. Some studies have shown that during boredom-inducing tasks, individuals may exert greater top-down control, particularly from frontal regions, to sustain attention on monotonous or repetitive content. This effortful engagement may manifest as increased beta band functional connectivity in related regions^55^. Gamma and beta node strength distribution across broad cortical areas highlights the global involvement of neural networks in distinguishing boredom from exciting states. The widespread gamma activity observed in frontal, parietal, temporal, and occipital regions likely reflects a high degree of network integration, facilitating the coordination of cognitive and affective processes necessary to endure boredom^57^. In contrast, the beta band findings indicate regionally focused connectivity patterns, potentially linked to cognitive regulation and attentional modulation during low-engagement states.^56^ Aside from these high-frequency bands, no significant differences were found in the node strengths of delta or theta bands. The alpha band showed moderate node strength, primarily in centroparietal regions. This is in line with the established role of alpha oscillations in inhibitory control, idling states, and relaxed wakefulness, which may be less directly engaged during boredom processing. Our results highlight the predominant involvement of gamma band node strength, suggesting that boredom and excitement impose considerable neural demands. The consistent engagement of high-frequency oscillations such as gamma and beta may reflect the cortical effort required to regulate attention and cognitive state during low-stimulation environments, contributing to the subjective experience of effort or discomfort often reported during boredom^54^.

Coherence network measures in the gamma band showed prominent differences compared to those in other EEG frequency bands (see Fig. 7). Specifically, during boredom, the clustering coefficient and efficiency (both global and local) were significantly higher, while the characteristic path length was shorter compared to excitement emotion processing. These findings provide valuable insights into how the brain’s functional networks reorganize during disengaging or low-stimulation states. The gamma band is known to play a big role in important brain functions like attention, memory, and integrating information from different senses. The observed increase in the clustering coefficient during boredom is due to the formation of more locally connected subnetworks, which may facilitate efficient local processing^23,41^. Similarly, increased network efficiency, increased network efficiency reflects more streamlined communication across distributed brain regions, potentially supporting internal cognitive processes that help individuals cope with repetitive or unstimulating environments. Together, these gamma-band dynamics may represent the brain’s adaptive attempt to maintain internal engagement without external stimulation.

Another important finding of the present study is the shorter characteristic path length observed during boredom. In graph theory, path length refers to the number of connections (or steps) required for information to travel from one node (brain region) to another within the network. A shorter path length indicates a more efficient and streamlined communication structure, allowing faster integration of information across distributed brain regions^41^. This mechanism may help the brain remain functionally engaged despite the lack of external stimulation, thereby preventing a complete shift into inattentiveness or disengagement. These findings support the view that boredom is not a passive or idle state, but rather one that engages active cognitive mechanisms^50,58^. The observed increases in connectivity and network efficiency during boredom suggest a functional reorganization of brain networks to compensate for reduced external input. In contrast, during excitement emotional processing, these network measures may not exhibit the same degree of optimization, possibly reflecting a more relaxed or externally guided cognitive state that requires less internal compensatory effort.

In addition to gamma band findings, we observed significant differences in the beta and alpha frequency bands during boredom and excitement emotion stimulus processing. Specifically, characteristic path length and global efficiency differed significantly in both bands. Notably, a pattern similar to the gamma band was seen in the shortened path length and increased global efficiency in the alpha and beta bands during boredom. Global efficiency quantifies the brain’s ability to quickly exchange information across the network, where higher values indicate more efficient communication between distant brain regions. The increased global efficiency observed in the alpha and beta bands may reflect the brain’s adaptive reconfiguration to preserve functional connectivity despite reduced external stimulation. In particular, the beta band, commonly associated with cognitive control^59^ and goal-directed processing^60^ may indicate the brain’s effort to sustain attentional and cognitive performance during a monotonous task. This could serve as a compensatory mechanism to counteract disengagement. Similarly, the alpha band, typically linked to inhibitory control, relaxed wakefulness, and reduced sensory input^61^, may reflect a passive processing mode in which the brain optimizes resource allocation to manage boredom with minimal cognitive demand. These alpha and beta network dynamics changes suggest that boredom elicits distinct frequency-specific adaptations in the brain’s functional architecture, supporting both compensatory cognitive control and low-arousal processing states.

From a learning perspective, these findings suggest that boredom may play a more complex and dynamic role in cognitive processing and the traditionally assumed^62^. Although boredom is typically viewed as a hindrance to learning, the observed increases in gamma and beta band activity during boredom may reflect active neural engagement aimed at managing reduced external stimulation^57^. In educational contexts, efficiently processing information is critical for meaningful learning. Previous studies have shown that enhanced gamma activity is associated with improved learning outcomes, as gamma oscillations support neural synchrony across networks involved in attention, perception, and memory formation^63^. In contrast, when learners encounter monotonous or unengaging content, a reduction in gamma coherence has been linked to less effective communication among brain regions responsible for focus and cognitive integration. Thus, boredom might function as a neural signal, prompting the brain to seek new sources of stimulation, alternative problem-solving strategies, or deeper levels of cognitive engagement^62^. If such states promote more efficient reorganization of brain networks, boredom could—paradoxically—enhance learning by stimulating adaptive changes in executive and attentional systems. Moreover, the significant differences in connectivity measures observed in the gamma, beta, and alpha bands during boredom may indicate an increased efficiency in neural communication within networks implicated in executive control, sustained attention, and working memory^57,64,65^. These dynamics may be particularly relevant in learning scenarios that require prolonged concentration or higher-order problem-solving. While prolonged or unmanaged boredom can impair attention and motivation, our results suggest that brief episodes of boredom may trigger cognitive reorganization, enabling learners to re-engage with material more deeply. In this sense, boredom could act not purely as a barrier to learning but as a potential catalyst for cognitive growth, encouraging the brain to arrange internal resources when external stimuli are insufficient. This interpretation opens new avenues for exploring how neural adaptability during boredom might be leveraged in educational design and learner support strategies.

### Limitations and future work

The present study includes some limitations. Firstly, the study is performed to understand the functional connectivity of boredom elicited by a validated video stimulus in a lab setting. Generalizing, the neural finding for deeper boredom in authentic learning contexts (e.g., the classroom) needs further studies. Moreover, only one elicitation stimulus was used to evoke boredom. To gain a deeper understanding of boredom, using different elicitation content in future work would allow us to examine various facets of boredom and its diverse manifestations. Furthermore, we could not analyze correlations between subjective video viewing durations, including early terminations, and the self-reported emotional responses. The SAQ was designed to capture participants’ peak emotional intensity during the videos, regardless of viewing duration. Consequently, the video duration data did not reliably reflect participants’ emotional engagement, limiting our ability to examine this relationship. Future studies could benefit from incorporating more detailed measures of engagement time and continuous emotional tracking to better understand how viewing duration influences affective responses. Additionally, this study focused on only four network measures, so future research should consider analyzing other global and local graph metrics. The use of spectral coherence to estimate functional connectivity may include spurious zero-lag correlations due to volume conduction. Future studies could consider incorporating variations of coherence measures, such as imaginary coherence (iCoh), to further refine functional connectivity estimates and validate the current findings. Lastly, future studies to explore and compare additional connectivity approaches (e.g., Directed Transfer Function, partial directed coherence, Granger causality) to provide a more comprehensive understanding of brain network dynamics, as these methods capture directionality and causal relationships that coherence alone may not fully reveal.

### Study implications

The implications of studying boredom’s functional connectivity are significant for education and mental health. For example, understanding the neural basis of boredom can help educators design more engaging and stimulating learning environments in educational settings. By identifying which brain regions are underactive or overactive during boredom, educators could tailor lessons that better align with students’ cognitive and emotional needs, helping to reduce disengagement and improve learning outcomes. Moreover, it may guide the development of personalized interventions, such as gamification or adaptive learning technologies, that target specific brain networks associated with attention and motivation. The observed elevation in gamma-band connectivity, particularly in regions associated with attention and cognitive control, may reflect the brain’s compensatory attempt to maintain engagement in emotionally neutral or low-stimulation environments. Interventions that strategically activate or support these networks could help sustain attention and motivation, even during monotonous or repetitive learning tasks. Additionally, boredom’s functional connectivity study has the potential to improve our understanding of broader cognitive processes, such as creativity and decision-making. As boredom often triggers mind-wandering and self-reflection, it may also influence problem-solving and creative thinking. By examining how the brain’s networks behave during these moments, researchers can uncover the neural mechanisms that drive creativity and explore ways to harness boredom as a tool for innovation.

## Conclusion

The present study analyzed the functional connectivity and the changes in the parameters of functional brain networks during the boredom state using EEG. Our results showed significant differences in the gamma, beta, and alpha bands when comparing the connectivity measures of boredom and excitement stimuli. Although boredom is typically regarded as a negative state associated with disengagement, our results suggest that boredom might actually activate important brain processes that help improve brain connections and thinking efficiency. The increase in gamma and beta brain activity during boredom may help the brain use its resources better, which can support learning. These findings challenge the common belief that boredom only gets in the way of learning and show that, when handled properly, boredom can actually help improve thinking and learning.

## Supplementary Information

Below is the link to the electronic supplementary material.

Supplementary Material 1

## Data Availability

The authors do not have permission to share the data. However, data access requests may be directed to the corresponding author for consideration.

## Declarations

### Generative AI and AI-assisted technologies in the writing process

During the preparation of this work, the author(s) used ChatGPT to assist with grammar improvement and language refinement. After using this tool, the author(s) reviewed and edited the content as needed to ensure accuracy, originality, and adherence to the intended academic and ethical standards.
