# Supplementary material for: EEG-based functional connectivity patterns during boredom in an educational context
Source: Sci Rep. 2025 Oct 10;15:35439. doi: 10.1038/s41598-025-19245-7 (PMC12514232; doi:10.1038/s41598-025-19245-7)
Supplement: Supplementary file 1 — Supplementary Material 1 [file 41598_2025_19245_MOESM1_ESM.pdf]

Appendix 1: State Affect Questionnaire (SAQ) used in this study

State Affect Questionnaire

Video 2

1. What emotion (choose one) did you feel while watching the video clip (please tick)?
   ☐ excitement ☐ disgust ☐ neutral ☐ boredom ☐ distress
2. Using the below scale, please indicate the intensity of the emotion you reported above during the video.

0	1	2	3	4	5	6	7	8
Not at all/none				Somewhat				Extremely

3. Did you feel any other emotion during the video clip?
   ☐ Yes ☐ No

   If so, what is [was] the emotion? ___________________

   How much of this emotion do [did] you feel (using the above scale)? _________
4. Have you seen this video before?
   ☐ Yes ☐ No
5. Did you close your eyes or look away during the video?
   ☐ Yes ☐ No
6. Did your mind wander, or did you think about things other than the video while watching it?
   ☐ Yes ☐ No
7. Overall feedback:

State Affect Questionnaire

Video 1

1. What emotion (choose one) did you feel while watching the video clip (please tick)?
   ☐ excitement ☐ disgust ☐ neutral ☐ boredom ☐ distress
2. Using the below scale, please indicate the intensity of the emotion you reported above during the video.

0	1	2	3	4	5	6	7	8
Not at all/none				Somewhat				Extremely

3. Did you feel any other emotion during the video clip?
   ☐ Yes ☐ No

   If so, what is [was] the emotion? ___________________

   How much of this emotion do [did] you feel (using the above scale)? _________
4. Have you seen this video before?
   ☐ Yes ☐ No
5. Did you close your eyes or look away during the video?
   ☐ Yes ☐ No
6. Did your mind wander, or did you think about things other than the video while watching it?
   ☐ Yes ☐ No
